# Effect of a Combination of Citrus Flavones and Flavanones and Olive Polyphenols for the Reduction of Cardiovascular Disease Risk: An Exploratory Randomized, Double-Blind, Placebo-Controlled Study in Healthy Subjects

**DOI:** 10.3390/nu12051475

**Published:** 2020-05-19

**Authors:** Maravillas Sánchez Macarro, Juan Pablo Martínez Rodríguez, Enrique Bernal Morell, Silvia Pérez-Piñero, Desirée Victoria-Montesinos, Ana María García-Muñoz, Fernando Cánovas García, Julián Castillo Sánchez, Francisco Javier López-Román

**Affiliations:** 1 Health Sciences Department, Universidad Católica San Antonio de Murcia (UCAM), Campus de los Jerónimos, E-30107 Murcia, Spain; sperez2@ucam.edu (S.P.-P.); dvictoria@ucam.edu (D.V.-M.); amgarcia13@ucam.edu (A.M.G.-M.); fcanovas@ucam.edu (F.C.G.); jlroman@ucam.edu (F.J.L.-R.); 2 Health Sciences Ph.D. Program, Universidad Católica San Antonio de Murcia (UCAM), Campus de los Jerónimos, E-30107 Murcia, Spain; laboratorio1@nutrafur.com; 3 Research and Development Department, Nutrafur iff-Health, E-30820 Murcia, Spain; j.castillo@nutrafur.com; 4 Unidad de Enfermedades Infecciosas, Hospital General Universitario Reina Sofia, E-30003 Murcia, Spain; ebm.hgurs@gmail.com; 5 Food Technology & Nutrition Department, Universidad Católica San Antonio de Murcia (UCAM), E-30107 Murcia, Spain

**Keywords:** bitter orange, cardiovascular risk, citrus flavonoids, endothelial function, grapefruit, olive, polyphenols, secoiridoids

## Abstract

A single-center, randomized, double-blind controlled trial was conducted to assess the efficacy of a food supplement based on a combination of grapefruit, bitter orange, and olive extracts administered for eight weeks (n = 51) versus placebo (n = 45) on reduction of cardiovascular risk in healthy volunteers. Study variables included flow-mediated vasodilation (FMD), blood pressure (BP), lipid profile, thrombotic status, oxidative stress biomarkers, inflammation-related biomarkers, anthropometric variables, quality of life, and physical activity. The per-protocol data set was analyzed. In the active product group, there were statistically significant within-group differences at eight weeks as compared with baseline in FMD, systolic and diastolic BP, total cholesterol, LDL-C, LDL-oxidase, oxidized/reduced glutathione ratio, protein carbonyl, and IL-6. Significant between-group differences in these variables were also found. Significant changes in anthropometric variables and quality of life were not observed in the study groups. Changes in the level of physical activity were not recorded. Treatment with the active product was well tolerated. All these findings, taken together, support a beneficial effect of supplementation with a mixture of grapefruit, bitter orange fruits, and olive leaf extracts on underlying mechanisms that may interact each other to decrease the cardiovascular risk in healthy people.

## 1. Introduction

Cardiovascular disease (CVD) is a major public health problem contributing substantially to morbidity, mortality, and escalating health care costs throughout the world. In the 2019 update report from the American Heart Association (AHA), the prevalence of CVD in adults ≥ 20 years was 48% overall and increased with advancing age both in men and women [1]. Among 10 leading causes that accounted for 74.1% of all registered deaths, heart diseases ranked first [1]. In the 2017 European cardiovascular statistics, CVD accounted for 45% of all deaths in Europe, being the main cause of death in men in all but 12 countries and the main cause of death in women in all but two countries [2]. Although age-adjusted mortality rates of CVD have declined over the past decades in high-income countries due to treatment advances and preventive measures [3], recent increases in the incidence of deaths from coronary heart disease and stroke offset the gains made in other disorders because of the aging society and the increased prevalence of diabetes and obesity [4].

There is consistent evidence of the contribution of major modifiable risk factors to the growing global burden of CVD worldwide, including tobacco smoke, diabetes, high cholesterol or hyperlipidemia, raised blood pressure, physical inactivity, obesity and overweight, alcohol use, and unhealthy diet [5,6,7,8]. Moreover, these conditions that are part of the CVD continuum often overlap. The role of diet is crucial in the development and prevention of CVD, and epidemiological studies have shown increased cardiovascular risk associated with high intakes of processed meat, sugar-sweetened beverages, highly processed carbohydrates, trans fats and salt, and low intakes of fruits and vegetables, whole grains, legumes, nuts and seeds, fish and fiber [9,10,11,12,13,14,15]. There is a high level of evidence (evidence A) of the favorable effects of low-fat diets, low-carbohydrate diets, the dash diet to lower blood pressure and the Mediterranean diet in people at high cardiovascular risk [16,17,18]. In fact, nutrition and dietary recommendations have been systematically included in clinical practice guidelines on primary prevention of CVD [19,20,21].

However, despite compliance with these recommendations, dietary intake of flavonoids in general and other relating polyphenolic compounds is really modest. Epidemiological studies have shown that the habitual intake of flavonoids in the range of 20–40 mg/day is below requirements to establish unequivocally a preventive-dose relationship [22,23]. This fact is even more significant for citrus flavonoids and olive polyphenols, given that the main sources of these compounds, citrus peels and olive leaves, are not used as ingredients in our diet. Consequently, the use of nutritional supplements becomes recommendable and almost necessary, both to allow access to ingestion of these active compounds and to ensure appropriate effective doses.

Nutritional supplements have also been a focus of increasing interest for CVD prevention based on increasing knowledge of biologic mechanisms to explain the health effects of multiple bioactive substances found in natural products [24,25,26,27,28]. In relation to the broad biological activities of a large group of naturally occurring polyphenols and the flavonoid class, many in vitro and in vivo studies reported health promoting actions ascribed to their antioxidant, antiatherogenic, anti-inflammatory, antithrombotic, antiplatelet, antiproliferative, vasodilatory, and vascular protective effects, all of which are beneficial properties for the underlying pathophysiological mechanisms of CVD [28,29,30,31,32,33,34,35]. In a multicenter trial in Spain, a Mediterranean diet supplemented with extra-virgin olive oil or nuts was associated with a significant reduction in the incidence of major cardiovascular events [36]. Other randomized controlled trials have shown reductions in oxidative stress markers and improvement in lipid and plasma antioxidant profiles with virgin olive supplements and concentrated/standardized extracts of the phenolic components of olive oil [37,38,39]. A number of beneficial effects of the consumption of dietary flavonoids found in foods, such as cocoa, apples, tea, citrus fruits, and berries on high blood pressure and endothelial dysfunction have been reported, and these effects are thought to have a significant impact upon vascular health [40,41,42].

Most multifactorial pathologies, particularly cardiovascular disorders, are the result of an imbalance of the set of responsible processes in organisms maintaining a situation of equilibrium or homeostasis that allow for adaptation to changes in the environment. The ability of organisms to maintain homeostasis is known as resilience. Therefore, enhancing resilience is an effective and innovative way to prevent the onset of pathologies or multifactorial situations. This approach represents an important innovation since it involves combining a set of bioactive ingredients in the same food that act simultaneously on the set of targets involved in the maintenance of homeostasis. This approach allows for developing new products more effective for the prevention of pathologies or multifactorial situations. Now, this strategy is also valuated in pharmaceutical treatments, considering the new evidences and endorsement of simultaneously targeting multiple risk factors for the prevention of CVD events using a specific approach with fixed-dose combinations of cholesterol- and blood pressure-lowering drugs, also known as polypills [43].

It is important to note that endothelial dysfunction is currently considered one of the first manifestations of vascular disease, mainly based on the concept of an imbalance in the bioavailability of active substances of endothelial origin, predisposing to inflammation, vasoconstriction, and increased vascular permeability, and being a marker that integrates the vascular risk of CDV patients.

The aim of the present study was to investigate the efficacy of supplementation with a combination of citrus flavones and flavanones and olive polyphenols on the reduction of cardiovascular risk in healthy subjects. A distinctive feature of the study was the large number of variables assessed, including endothelial function, lipid metabolism, blood pressure, thrombotic and antioxidant status, inflammation, anthropometric measurements, lifestyle, and quality of life.

## 2. Materials and Methods

### 2.1. Study Design

The study was a single-center, randomized, parallel, double-blind, placebo-controlled trial that was conducted at the Health Sciences Department of the Saint Anthony Catholic University (Murcia, Spain). The primary objective of the study was to assess the efficacy of an 8-week daily regimen with a dietary supplement obtained from the combination of grapefruit and bitter orange immature fruits and olive leaf extracts on the reduction of cardiovascular risk assessed by flow-mediated vasodilation (FMD) as a measure of endothelial function. Secondary objectives included assessment of changes of endothelial function, lipid profile, blood pressure, thrombotic and antioxidant status, inflammatory biomarkers, anthropometric measures, and quality of life, as well as safety of treatment.

The trial was performed between October 2016 and December 2018. The study protocol was approved by the Institutional Review Board of the Saint Anthony Catholic University (Murcia, Spain) and was registered in the ClinicalTrials.gov. (NCT04114916) in April, 2018. The study was conducted in accordance with the Declaration of Helsinki. All participants gave written informed consent. Participants’ records and information were anonymized.

### 2.2. Participant Selection and Randomization

Healthy Caucasian male and female volunteers aged 40 to 75 years old were recruited by disseminating information on the study through talks in women’s centers, senior centers, neighborhood associations, and mass media. Inclusion criteria were as follows: fasting serum total cholesterol ≥ 180 mg/dL and low-density lipoprotein cholesterol (LDL-C) ≥ 110 mg/dL; body mass index (BMI) between 18.5 and 34.9 kg/m^2^; serum hemoglobin > 120 g/L in men and > 110 g/L in women; hematocrit > 40% in men and > 35% in women; platelet count > 170 × 10^9^/L; and postmenopausal status. Subjects were excluded in the presence of the following conditions: thyroid dysfunction, infection, or any type of chronic disease; history an ischemic vascular event in the last months or major surgery within 3 months before enrolment; current treatment with niacin or fibrates, drugs and/or nutraceutical products for hypertension, diabetes or hyperlipidemia, drugs that due to their narrow therapeutic margin would require monitoring of plasma drug levels, or drugs that affect body weight and/or appetite; blood donation (≥0.5 L) in the last month; use of omega-3 and/or omega-6 polyunsaturated fatty acids supplements in the last 3 months; vegetarian diet or willingness to follow a diet during the study; alcohol abuse or consumption of >3 glasses of wine/beer per day; participation in other clinical studies within the prior 3 months; and history of hypersensitivity or poor tolerance to any component of the study product. Subjects who were judged as ineligible by the investigators for other reasons were excluded, as were smokers or non-smokers who modify their smoking habit during the course of the study.

Participants were randomly assigned in a 1:1 ratio to a dietary intervention with the nutritional supplement or supplementation with placebo. Randomization was performed by means of a computer-generated random number sequence by an independent investigator.

### 2.3. Intervention and Study Procedures

Participants were administered the active product (yellowish dry powder) or placebo (yellowish dry powder maltodextrin) during the 8 weeks. The active product was a blend compound (Citroven™) obtained from Nutrafur iff-Health (Murcia, Spain) based on the combination of two hydroethanolic extracts from immature citrus fruits, grapefruit (*Citrus paradisi* Macfad, naringin, narirutin, rhoifolin, poncirin, apigenin, etc.) and bitter orange (*Citrus aurantium* L., naringin, neohesperidin, neodiosmin, luteolin, etc.), and one hydroethanolic extract from an olive leaf (*Olea europaea* L., olive secoiridois, oleuropein family, hydroxytyrosol, etc.). Participants were instructed to take two capsules a day (2 × 500 mg each) of the study supplement in the morning and in the evening, 12 h apart, either with meals or between meals. The total daily intake of the active compounds was 250–300 mg of flavanone-glycosides (naringin, neohesperidin, narirutin, poncirin, …), 175–200 mg of flavones (apigenin, luteolin, rhoifolin, neodiosmin, …), and 85–90 mg of olive polyphenols (secoiriodids and phenolics), mainly oleuropein.

In vivo acute oral toxicity of the compound has been previously evaluated according to guidelines of the Organization for Economic Co-operation and Development (OECD) (Test No. 421: Reproduction/Developmental Toxicity Screening Test). The study of acute oral toxicity of the product showed that the lethal dose LD_50_ for the product can be considered greater than 2000 mg/kg body weight (unpublished data). Taking into account these results and following OECD recommendations, the compound could be classified according to the Globally Harmonized System (GHS) as an “unclassified substance” or of very low toxicity (GHS category 5). Each individual ingredient of Citroven™ could be classified as GHS category 5 (of very low toxicity).

The study included two visits, one baseline visit and a final visit at 8 weeks. At the baseline visit, the written informed consent was obtained, fulfilment of the inclusion criteria was checked, and the study product was provided. Clinical assessments included detailed medical history, dietary survey, and evaluation of the quality of life, measurement of anthropometric variables, blood pressure, and brachial artery plethysmography. At the 8-week visit, the same variables were recorded. Compliance with the study product was checked by counting the returned capsules in the medication container. Adverse events were ascertained by directly asking the participant and from results of laboratory tests.

Venous blood samples were taken after 12 h fasting at each of the visits (initial and 8-week visit) for laboratory analysis. Participants were instructed to refrain from moderate-severe physical exercise for at least 24 h prior blood testing. During the study period, subjects were not allowed to change dietary habits, use plant sterol-enriched foods, and begin or modify any hormonal treatment as well as to take aspirin, ibuprofen, naproxen or any COX-2 inhibitor drug at least 24 h before each study visit.

### 2.4. Study Variables

Cardiovascular risk was assessed by measurement of FMD. FMD was assessed in the brachial artery using a high-resolution echo-Doppler ultrasound system (SonoSite MicroMaxx HFL38) with a 6–13 MHz linear array transducer according to guidelines of the International Brachial Artery Reactivity Task Force [44,45]. Briefly, first a sphygmomanometer blood pressure cuff was first placed above the antecubital fossa. A baseline resting image was acquired and measurements of the diameter of the brachial artery were taken. Thereafter, arterial occlusion was created by cuff inflation to suprasystolic pressure for 5 min. After 1 min of deflating the cuff, the new measurement was made. Results were expressed as percent of change of arterial diameter (mm) after occlusion in response to hyperemia over the baseline diameter, using the following equation: FMD = (peak of hyperemia diameter) − (baseline diameter)/(baseline diameter) × 100. Blood pressure was measured coinciding with blood withdrawal in the morning using an OMRON M6 AC blood pressure monitor (Omron Healthcare España). Subjects sat comfortably in a quiet environment for 5 min. Then, blood pressure was measured a total of three times, 2 min apart and the average of the last to measurement was calculated [46].

For biochemical analyses, blood samples were drawn at baseline and after completing 8 weeks of dietary supplementation or placebo. Laboratory studies included (a) lipid profile (cholesterol, LDL-C and high-density lipoprotein cholesterol [HDL-C]) using the clinical chemistry analyzer BA 400 Bioystems, and oxidized LDL by Human Oxidized LDL ELISA kit, Elabscience Biotechnology Inc., Houston, TX, USA); (b) thrombotic status (von Willebrand factor [vWF]), vWF levels were estimated quantitatively by the enzyme linked-fluorescent assay (ELFA method, Biomerieux, Spain); (c) oxidative stress biomarkers (reduced/oxidized glutathione ratio [GSH/GSSH ratio] by liquid chromatography-mass spectrometry, and protein carbonyl level by OxiSelect™ Protein Carbonyl ELISA kit, Cell Biolabs, Inc., San Diego, CA, USA); and (d) inflammation-related biomarkers (high sensitivity C-reactive protein [hs-CRP] by colorimetric using assay analyzer ILAB 600 [Instrumentation Laboratory], plasma levels of interleukin-6 [IL-6] by Interleukin-6 High Sensitivity ELISA kit, IBL International GmbH, Hamburg, Germany, and TNF-α levels by TNF-α High Sensitivity ELISA kit, IBL International GmbH).

Anthropometric variables included body weight, BMI, and free fat mass determined by bio-impedance analysis (BIA) on a whole body BIA analyzer Tanita BC-420MA (Tanita Corporation, Tokyo, Japan). Health-related quality of life was assessed using a Spanish validated version of the 12-item short form health survey questionnaire (SF-12) [47]. Lifestyle variables included a dietary recall interview of the previous 3 days before starting the nutritional supplement and for the last 72 h before the end of the 8-week consumption of the product. The level of physical activity was recorded using the Global Physical Activity Questionnaire (GPAQ) [48] and results expressed as MET-min/week.

Safety variables included blood cell count, alanine aminotransferase (ALT), aspartate aminotransferase (AST), bilirubin, lactate dehydrogenase (LDH), gamma-glutamyl transpeptidase (GGT), blood urea nitrogen, and serum creatinine levels.

### 2.5. Statistical Analysis

The per-protocol (PP) data set was analyzed; that is, all participants who completed the 8-week study period. Measurement of FMD was a primary outcome and the others were secondary outcomes. Categorical variables are expressed as frequencies and percentages and continuous variables as mean ± standard deviation (SD). The chi-square (χ^2^) test or the Fisher’s exact test were used for the comparison of categorical variables between the study groups, and the analysis of variance (ANOVA) for repeated measures with two study factors: within subject factor (time: baseline and final) and between-subject factor (intervention: active product and placebo) for paired data. The Tukey’s procedure or the Bonferroni’s method was used for pairwise comparisons.

Statistical analysis was performed with the SPSS version 21.0 (IBM Corp., Armonk, NY, USA). Statistical significance was set at *p* < 0.05.

## 3. Results

**Figure 1:**
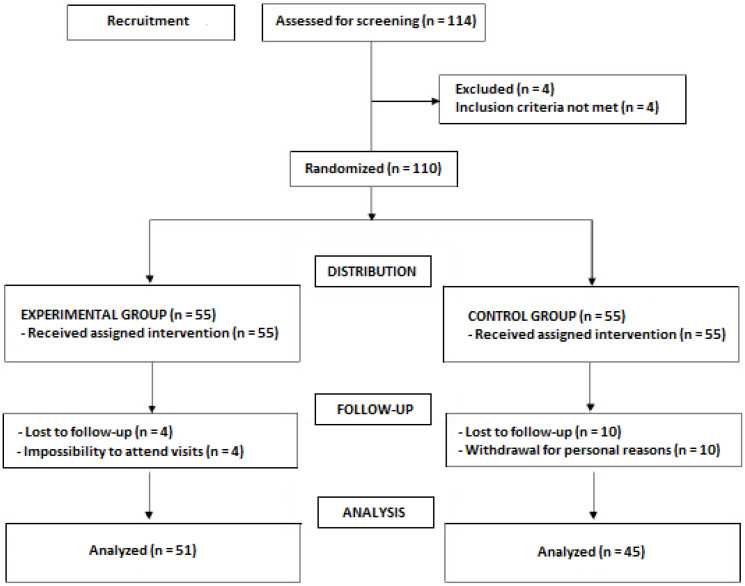
Flow chart of the study population.

A total of 114 subjects volunteered for the study but four of them did not meet the inclusion criteria and were excluded. The remaining 110 subjects were randomized (55 in each study group) but 14 were lost to follow-up. The final study population included 51 subjects in the active supplement group (27 men, 24 women; mean age 47.3 ± 11.2 years) and 45 in the placebo group (26 men, 19 women; mean age 51.3 ± 10.8 years) (Figure 1).

Results of the study variables at baseline and at the final visit are shown in Table 1.

The use of the active product over a period of eight weeks was associated with an improvement of FMD. The comparison of baseline (6.73 ± 5.27%) and final values (9.77 ± 5.56%) showed statistically significant differences (*p* < 0.001) in the active product group, whereas differences were not significant in the placebo group. Between-group differences in FMD were statistically significant (*p* = 0.039). Both systolic and diastolic blood pressure decreased significantly in the active product group, whereas changes were not significant among participants treated with placebo. Between-group differences were significant for diastolic blood pressure (*p* = 0.045).

In relation to the lipid profile, there was a significant improvement in serum levels of total cholesterol, LDL-C, and oxidized-LDL (ox-LDL), with statistically significant differences for within-group comparisons in subjects assigned to the active product group, as well as between group-differences versus placebo. Regarding variables related to oxidative stress, the GHS/GSSH ratio increased significantly in the active product treatment product only and protein carbonyl decreased significantly in the active product treatment group only. Between-group comparisons of these variables showed statistically significant differences (*p* = 0.003 and *p* = 0.012, respectively). In relation to inflammation-related biomarkers, serum levels of IL-6 decreased from 1.49 ± 0.96 pg/mL at baseline to 0.91 ± 0.56 pg/mL after eight weeks of treatment with the active product (*p* < 0.001), whereas changes in the placebo group were not significant. Between-group differences in serum levels of IL-6 during the study were statistically significant (*p* < 0.001). No significant differences were found in levels of the von Willebrand factor for any other group of the study.

Statistically significant changes in anthropometric variables, scores of the physical health, mental health, and total score of the SF-12 questionnaire were not found in the study groups. Significant changes in the level of physical activity were not recorded.

Treatment with the active product was well tolerated. Neither adverse events nor changes in cell blood count, liver enzymes, and serum levels of blood urea nitrogen and creatinine were observed.

## 4. Discussion

In the present 8-week study, daily consumption of a nutritional supplement based on a particular and specific combination of citrus flavones and flavanones and olive polyphenols was associated with beneficial changes in a number of CVD-related factors, including FMD, blood pressure and lipid profile, as well as biomarkers of antioxidant and anti-inflammatory status. These findings add evidence to the positive effects of polyphenols and flavonoids on the underlying mechanisms involved in cardiovascular damage.

Regarding the role of endothelial dysfunction in the development of CVD, a dysfunctional endothelium has an increased consumption of nitric oxide (NO), which creates favorable conditions for platelet plus leukocyte activation and adhesion, as well as the activation of cytokines that increase the permeability of the vessel wall to oxidized lipoproteins and inflammation mediators. This finally results in structural damage of the arterial wall with smooth muscle cell proliferation and atherosclerotic plaque formation [49]. Dietary flavonoids have also been shown to improve endothelial function and to lower blood pressure by causing vasodilation in the peripheral vasculature [16]. Several flavonoids increase the bioavailability of endothelial vasodilator factors, mainly NO and also endothelium-derived hyperpolarizing factor (EDHF) and inhibit the production of pro-inflammatory substances which considerably improves the function of vascular endothelium [31,32,35]. In the present study, a clear improvement of FMD was found among subjects assigned to the active product, with significant differences at the end of the study as compared with baseline and within group differences versus subjects treated with placebo. If FDM is considered as a predictive variable of cardiovascular risk [49,50,51], the use of this active product was associated with CDV risk reduction.

Similar intervention studies of the effect of active polyphenol extracts on endothelial dysfunction are scarce. The use of green tee catechins (GTC) in healthy smokers was associated with anti-atherosclerotic effects on dysfunctional vessels only in the group of high-dose GTC (500 mg/day), with no effect in the placebo group or in the 80 mg/day GTC dose [52]. In hypertensive subjects, a polyphenol-rich diet (50–100 mg/day) via consumption of fruit, vegetables, berries, and dark chocolate for eight weeks resulted in a significant improvement of endothelium-dependent vasodilator response [53]. In a cross-over study of healthy subjects, the consumption of a loading dose of black tee (400 mg flavonoids) and maintenance dose (130 mg) after 120 min had no effect on improving endothelial function, suggesting the need of interventions with a longer duration [54]. It has been shown that daily intake of 25 mg of pure epichatechin for two weeks did not reduce cardiometabolic risk factors in overweight-to-obese adults [55]. In another cross-over study, consumption of an apple flavanols extract (270 mg monomeric [epichatechin] and oligomeric [procyanidins] flavanols) for four weeks did not affect endothelial function [56]. These data indicate that citrus flavones and flavanones combined with olive secoiridoids appear to show a higher efficacy than the flavan-3-ol (catechins) family usually present in green tea, grapeseed, cacao, and apple.

Currently available evidence of the beneficial effect of an olive oil-enriched diet on blood pressure found in systematic reviews and meta-analyses [57,58,59] was also confirmed in our study. Modest decreases in blood pressure between 4% and 7% are explained by the healthy condition of participants.

The reduction of ox-LDL in the intervention group with a mean difference of −342.84 pg/mL as compared with baseline is a remarkable finding of the study. Other studies, however, have shown varied results. In a randomized double-blind trial, consumption of a standardized extract of maqui berry (anthocyanins 486 mg/day) for four weeks was associated with reduced levels of ox-LDL compared to baseline, but no differences were evident at 40 days of follow-up [60]. In another clinical trial, the consumption of an extract of low molecular weight procyanidins 150 mg/day for five years resulted in a decrease of −31.7 U/L in the intervention group, with a percentage of change versus placebo of −32.7% [61]. In patients with metabolic syndrome, supplementation with olive fruit extract (9–10 mg/day of hydroxytyrosol) combined with red yeast rice (10–11 mg/day of monacolins, including 5–6 mg/day of lovastatin) for eight weeks resulted in a 20% reduction of ox-LDL vs. an increase of 5% in the placebo group [62]. By contrast, ingestion of olive pomace-enriched biscuit (15–20 mg/day of hydroxytyrosol and derivatives) for eight weeks did not induce statistically significant changes in ox-LDL [63]. In the case of the two olive polyphenolic compounds, oleuropein and hydroxytyrosol, potentially responsible for the reduction of ox-LDL levels, the dose used in one study of 15–20 mg/day does not appear to be sufficient for real effectiveness [63], and the 20% decrease of ox-LDL reported in the other study [62] is lower than the 40–45% obtained with an intake of 80–90 mg/day in our study. Moreover, other components of the nutritional product, such as the citrus flavone luteolin, has been shown to inhibit oxidized LDL and TNF-α-induced VCAM-1 expression, as well as suppression of the IκBα/NF-κB signaling pathway [64]. Luteolin is capable of protecting the myocardium against ischemia-reperfusion injury, which is attributed to the actions of luteolin mediated through downregulation of NO production and its own antioxidant properties [65,66].

In relation to oxidative stress biomarkers, a 62–65% increase in GSH/GSSH ratio and a 30–32% reduction of the protein carbonyl level was obtained, which is consistent with a reduction of ox-LDL as biomarkers of cardiovascular risk reduction. A few intervention clinical trials have evaluated the GSH/GSSH ratio as a marker of antioxidant status. In a double blind, placebo-controlled crossover pilot study of type 2 diabetic patients at high cardiovascular risk, four weeks of treatment with an aged garlic extract (1200 mg/day of alliin and other sulfoxide compounds) had no significant effect on blood GSH/GSSG and did not improve endothelial function [67]. However, in another study [68] the use of grape seed extract (600 mg/day), which is a flavonoid-rich product in proanthocyanidins (450–500 mg/day), for four weeks was associated with similar increases of the GSH/GSSH ratio as those obtained in our study.

A remarkable finding of the study was a significant reduction of the cytokine IL-6, a marker of inflammation. High IL-6 concentrations have been associated with increased risk of myocardial infarction (MI) in healthy men. Moreover, IL-6 and its receptor levels have an early peak at the acute phase of MI, likely related to plaque instability [69]. Persistent inflammation has been proposed to contribute to various stages in the pathogenesis of cardiovascular disease, with IL-6 receptor signaling propagating downstream inflammation cascades [70]. In the Health, Aging, and Body Composition study of 2225 participants 70 to 79 years old without baseline cardiovascular disease, IL-6 was significantly associated with all outcomes, including coronary heart disease, stroke, and congestive heart failure [71]. In this respect, the contribution of the active supplement to reduce IL-6 levels (38–40%) after a short-term period of treatment is clinically relevant and, to our knowledge, reduction of plasma levels of IL-6 of this magnitude has not been previously reported in intervention clinical trials with nutritional supplements. Of note, the flavone apigenin could also be involved in the reduction of IL-6. Apigenin has been shown to exhibit several biological activities, including anti-inflammatory and antioxidant properties with a role in scavenging free radicals [25,72]. It has been shown that apigenin inhibits the lipopolysaccharide-induced inflammatory response through multiple mechanisms in macrophages, such as NF-κB, MAPK/ERK, and JNK pathways [73].

All of these improvements in FMD, blood pressure, lipid profile, antioxidant biomarkers, and IL-6 were unrelated to changes in potentially confounding variables, such as body weight, BMI, fat mass, or physical activity level.

The present results, however, should be interpreted by taking into account the limitations of the study, including the exploratory nature of the trial, the reduced sample size, and the treatment period of eight weeks only. It would be appropriate to test the use of the nutritional supplement in the long run.

## 5. Conclusions

This study shows that supplementation with a combination of two citrus fruit extracts, grapefruit (*Citrus paradisi* Macfad) and bitter orange (*Citrus aurantium* L.), and one olive leaf extract (*Olea europaea* L.) during eight weeks improved endothelial function measured by FMD, reduced blood pressure and lipid metabolism-related parameters, and improved antioxidant and inflammatory status. All these findings, taken together, suggest a beneficial effect of the nutritional supplement on underlying mechanisms that may interact each other to decrease the cardiovascular risk in healthy people. However, further randomized studies are needed to confirm the present results of this exploratory trial, in particular with a larger study population and consumption of the nutritional supplement over a longer period of time.

## Figures and Tables

**Table 1:** Results of the study variables before and after consumption of the nutritional supplement.

Variables	Study Groups						Between-Group *p*-Value (F Snedecor)
	Active Group (n = 51)			Placebo Group (n = 45)			
	Baseline (1) ± SD	8-Week (2) ± SD	Δ 2-1 (95% CI) *p*-Value	Baseline(1) ± SD	8-Week (2) ± SD	Δ 2-1 (95% CI) *p*-Value	
Flow mediated vasodilation (FMD), %	6.73 ± 5.27	9.77 ± 5.56	3.04 (1.448 to 4.617) **<0.001**	6.85 ± 4.56	7.39 ± 5.81	0.530 (−1.237 to 2.297) 0.636	**0.039** (F = 4.39)
Blood pressure, mm Hg							
Systolic	123.6 ± 13.8	118.7 ± 11.9	−4.89 (−7.7 to −2.06) **0.001**	122.8 ± 8.8	120.8 ± 11.6	−1.98 (−5.04 to 1.09) 0.203	0.168 (F = 1.94)
Diastolic	84.2 ± 9.2	81.7 ± 8.9	−2.49 (−4.88 to −0.01) **0.041**	79.7 ± 8.3	80.8 ± 7.8	1.12 (−1.46 to 3.70) 0.392	**0.045** (F = 4.15)
Laboratory tests							
Total cholesterol, mg/dL	242.2 ± 31.9	228.6 ± 29.6	−13.57 (−21.09 to −6.05) **0.001**	235.7 ± 38.9	242.0 ± 35.6	6.38 (−1.63 to 14.38) 0.117	**0.001** (F = 12.99)
LDL-C, mg/dL	144.2 ± 28.1	135.4 ± 26.6	−8.80 (−14.49 to −3.12) **0.003**	138.8 ± 31.3	139.9 ± 28.0	1.11 (−4.94 to 7.16) 0.717	**0.02** (F = 5.62)
HDL-C, mg/dL	63.6 ± 12.8	65.3 ± 13.1	1.65 (−0.82 to 4.11) 0.188	63.2 ± 15.8	62.1 ± 16.4	−1.09 (−3.71 to 1.54) 0.412	0.135 (F = 2.28)
Triglycerides, mg/dL	115.1 ± 54.0	115.5 ± 67.1	0.471 (−14.73 to 15.67) 0.951	118.9 ± 55.1	125.0 ± 74.1	6.02 (−10.16 to 27.20) 0.462	0.621 (F = 0.25)
Von Willebrand factor, %	120.9 ± 45.9	119.9 ± 36.7	0.833 −1.08 (−11.28 to 9.11)	119.5 ± 50.4	124.4 ± 46.0	4.95 (−6.04 to 15.94) 0.374	0.426 (F = 0.64)
LDL-oxidase, pg/mL	810.3 ± 555.4	467.5 ± 239.6	−342.84 (−459.68 to −226.00) **<0.001**	656.6 ± 402.3	692.2 ± 363.9	35.66 (−88.72 to 160.05) 0.571	**<0.001** (F = 19.39)
Oxidized Glutathione (GSSH), nmol/mg protein	0.47 ± 0.095	0.35 ± 1.59	−0.12 (−0.19 to −0.06) **<0.001**	0.52 ± 0.092	0.48 ± 0.11	-0.04(−0.11 to 0.04) 0.345	0.081 (F = 3.16)
Reduced Glutathione (GSH), nmol/mg protein	22.6 ± 6.0	23.6 ± 3.0	0.99 (−1.08 to 3.06) 0.342	21.9 ± 3.1	22.1 ± 3.1	0.20 (−2.15 to 2.56) 0.864	0.617 (F = 0.25)
GSH/GSSH, ratio	49.8 ± 15.4	81.9 ± 33.8	32.02 (19.90 to 44.15) **<0.001**	43.8 ± 11.9	47.6 ± 10.9	3.8 (−9.96 to 17.60) 0.580	**0.003** (F = 9.50)
Protein carbonyl, nmol/ mg protein	0.94 ± 0.48	0.65 ± 0.29	−0.29 (−0.45 to −0.13)) **0.001**	0.77 ± 0.54	0.79 ± 0.44	0.013 (−0.16 to 0.19) 0.881	**0.012** (F = 6.52)
C-reactive protein, mg/L	2.53 ± 3.07	2.35 ± 2.61	−0.18 (−0.94 to 0.58) 0.641	2.06 ± 2.50	1.91 ± 1.87	−0.15 (−0.97 to 0.67) 0.716	0.960 (F = 0.003)
Interleukin-6 (IL-6), pg/mL	1.49 ± 0.96	0.91 ± 0.56	−0.57 (−0.79 to −0.35) **<0.001**	1.38 ± 1.82	1.52 ± 2.24	0.14(−0.10 to 0.37) 0.251	**<0.001** (F = 18.78)
TNF-α, pg/mL	6.82 ± 0.64	6.63 ± 1.04	−0.19(−0.51 to 0.13) 0.250	6.96 ± 0.61	6.97 ± 0.81	0.01 (−0.34 to 0.35) 0.974	0.417 (F = 0.66)
Body weight, kg	76.8 ± 13.9	76.7 ± 14.6	−0.15 (−0.90 to 0.60) 0.695	76.7 ± 14.8	76.8 ± 14.9	0.06 (−0.74 to 0.86) 0.891	0.712 (F = 0.14)
Body mass index, kg/m^2^	27.2 ± 4.2	27.1 ± 4.2	−0.08 (−0.24 to 0.07) 0.286	26.5 ± 3.9	26.5 ± 3.8	0.03 (−0.13 to 0.20) 0.711	0.317 (F = 1.01)
Fat mass, kg	25.4 ± 11.7	25.1 ± 11.4	−0.32 (−2.36 to 1.71) 0.752	21.8 ± 7.5	22.1 ± 8.0	0.31 (−1.82 to 2.43) 0.775	0.671 (F = 0.18)
SF-12 questionnaire							
Physical health, score	76.6 ± 17.3	79.0 ± 16.8	2.38 (−0.98 to 5.74) 0.163	82.2 ± 11.1	82.8 ± 11.9	0.66 (−3.00 to 4.32)) 0.721	0.493 (F = 0.47)
Mental health, score	75.0 ± 17.1	77.7 ± 17.2	2.71 (−1.17 to 6.59) 0.168	78.5 ± 12.5	78.5 ± 13.8	−0.05 (−4.27 to 4.17) 0.980	0.340 (F = 0.92)
Total score	75.7 ± 15.7	77.9 ± 15.1	2.2 (−0.89 to 5.29) 0.161	80.7 ± 9.9	80.2 ± 10.5	−0.5 (−3.87 to 2.87) 0.768	0.243 (F = 1.38)
Physical activity, MET-min/week	3125.2 ± 3582.1	3004.3 ± 3254.1	−120.9 (−738.1 to 496.4) 0.698	3260.6 ± 3261.0	3254.5 ± 3121.7	−6.1 (−734.8 to 722.7) 0.987	0.811 (F = 0.06)

Data are reported as mean and SD of baseline and after 8 weeks of product and placebo consumption. Δ 2-1 represents the difference between means for those two groups (95% confidence interval). *p*-value between-groups in ANOVA for repeated measures with two study factors (time × group) is also reported. Significant values are in bold.
